# A new version of Carleson measure associated with Hermite operator

**DOI:** 10.1186/s13660-018-1771-2

**Published:** 2018-07-16

**Authors:** Jizheng Huang, Yaqiong Wang, Weiwei Li

**Affiliations:** 1 School of Sciences, Beijing University of Posts and Telecommunications, Beijing, China; 2 College of Sciences, North China University of Technology, Beijing, China

**Keywords:** 42B30, 42B35, Hermite operator, Carleson measure, Dual space, Hardy space, BMO space

## Abstract

Let $L=-\Delta+|x|^{2}$ be a Hermite operator, where Δ is the Laplacian on $\mathbb {R}^{d}$. In this paper we define a new version of Carleson measure associated with Hermite operator, which is adapted to the operator *L*. Then, we will use it to characterize the dual spaces and predual spaces of the Hardy spaces $H_{L}^{p}(\mathbb {R}^{d})$ associated with *L*.

## Introduction

In recent years, the study of function spaces associated with Hermite operators has inspired great interest. Dziubański [7] introduced the Hardy space $H_{L}^{p}(\mathbb {R}^{d})$, $0< p\leq1$, by using the heat maximal function and established its atomic characterization. Dziubański et al. [8] and Yang et al. [20] introduced and studied some BMO spaces and Morrey–Campanato spaces associated with operators. Deng et al. [5] introduced the space $\mathit{VMO}_{L}(\mathbb {R}^{d})$ and proved that $(\mathit{VMO}_{L}(\mathbb {R}^{d}))^{*}=H_{L}^{1}(\mathbb {R}^{d})$. Moreover, recently, Jiang et al. in [14] defined the predual spaces of Banach completions of Orlicz–Hardy spaces associated with operators. Bui et al. [3] considered the Besov and Triebel–Lizorkin spaces associated with Hermite operators.

One of the main purposes of studying the function spaces is to give the equivalent characterizations of them, for example, square functions characterizations for Hardy spaces [10], Carleson measure characterizations for BMO spaces [8] or Morry–Campanato spaces [6]. The aim of this paper is to give characterizations of the dual spaces and predual spaces of the Hardy spaces $H_{L}^{p}(\mathbb {R}^{d})$ by a new version of Carleson measure. Now, let us review some known facts about the function spaces for *L*.

Let *L* be the basic Schrödinger operator in $\mathbb {R}^{d}$, $d\geq 1$, the harmonic oscillator $L=-\Delta+|x|^{2}$. Let $\{T_{t}^{L}\}_{t>0}$ be a semigroup of linear operators generated by −*L* and $K_{t}^{L}(x,y)$ be their kernels. The Feynman–Kac formula implies that

1 $$ 0\leq K_{t}^{L}(x,y)\leq\widetilde{T_{t}}(x,y)=(4 \pi t)^{-\frac{d}{2}}\exp \biggl(-\frac{|x-y|^{2}}{4t} \biggr). $$

Dziubański [7] defined Hardy space $H_{L}^{p}(\mathbb{R}^{d})$, $0< p\leq1$ as

$$H_{L}^{p}\bigl(\mathbb{R}^{d}\bigr)= \bigl\{ f\in \mathcal{S}'\bigl(\mathbb{R}^{d}\bigr): Mf\in L^{p}\bigl(\mathbb{R}^{d}\bigr) \bigr\} , $$

where

$$Mf(x)=\sup_{t>0} \bigl\vert T_{t}^{L}f(x) \bigr\vert . $$

The norm of Hardy space $H_{L}^{p}(\mathbb{R}^{d})$ is defined by $\|f\|_{H_{L}^{p}}=\|Mf\|_{L^{p}}$.

### Remark 1

For simplicity, we just consider the case of $\frac{d}{d+1}< p\leq1$ in this paper. But all of our results hold for $0< p\leq1$.

Let $\rho(x)=\frac{1}{1+|x|}$ be the auxiliary function defined in [17]. This auxiliary function plays an important role in the estimates of the operators and in the description of the spaces associated with *L*. Then, for $\frac{d}{d+1}< p\leq1$ and $1\leq q\leq\infty$, a function *a* is an $H_{L}^{p, q}$-atom for the Hardy space $H_{L}^{p}(\mathbb {R}^{d})$ associated with a ball $B(x_{0},r)$ if

$$\begin{aligned} (1)&\quad \operatorname{supp} a\subset B(x_{0},r), \\ (2)&\quad \|a\|_{L^{q}}\leq \bigl\vert B(x_{0},r) \bigr\vert ^{\frac{1}{q}-\frac{1}{p}}, \\ (3)&\quad \mbox{if }r< \rho(x_{0}), \mbox{then } \int a(x)\,dx=0. \end{aligned}$$

The atomic quasi-norm in $H_{L}^{p}(\mathbb {R}^{d})$ is defined by

$$\|f\|_{L\text{-atom},q}=\inf \Bigl\{ \Bigl(\sum|c_{j}|^{p} \Bigr)^{1/p} \Bigr\} , $$

where the infimum is taken over all decompositions $f=\sum c_{j}a_{j}$ and $a_{j}$ are $H_{L}^{p, q}$-atoms.

The atomic decomposition for $H_{L}^{p}(\mathbb{R}^{d})$ is as follows (see [7]).

### Proposition 1

*Let* $\frac{d}{d+1}< p\leq1$, *we have that the norms* $\|f\|_{H_{L}^{p}}$ *and* $\|f\|_{L\textit{-}\mathrm{atom},q}$ *are equivalent*, *that is*, *there exists a constant* $C>0$ *such that*

$$C^{-1}\|f\|_{H_{L}^{p}}\leq\|f\|_{L\textit{-}\mathrm{atom},q}\leq C \|f \|_{H_{L}^{p}}, $$

*where* $1\leq q\leq\infty$.

We define Campanato space associated with *L* as (cf. [1] or [20]).

### Definition 1

Let $0\leq\alpha<1$, a locally integrable function *g* on $\mathbb {R}^{d}$ belongs to $\Lambda_{\alpha}^{L}$ if and only if $\|g\|_{\Lambda_{\alpha}^{L}}<\infty$, where

$$ \|g\|_{\Lambda_{\alpha}^{L}}= \sup_{B\subset\mathbb {R}^{d}} \biggl\{ |B |^{-\frac{\alpha}{d}} \biggl( \int_{B} \bigl\vert g-g(B, x_{0}) \bigr\vert ^{2}\frac{dx}{|B|} \biggr)^{1/2} \biggr\} $$

and

$$ g(B,x_{0})= \textstyle\begin{cases} \frac{1}{ | B(x_{0},r) |} \int_{B(x_{0},r)} g(y) \,dy, &\mbox{if } r< \rho(x_{0}), \\ 0, &\mbox{if } r \geq\rho(x_{0}). \end{cases} $$

The duality of $H_{L}^{p}(\mathbb {R}^{d})$ and $\Lambda_{d(1/p-1)}^{L}$ can be found in [12] or [20].

In order to give the Carleson measure characterization of $\Lambda _{d(1/p-1)}^{L}$, we need some notations of the tent spaces (cf. [4]).

Let $0< p<\infty$ and $1\leq q\leq\infty$. Then the tent space $T^{p}_{q}$ is defined as the space of functions *f* on $\mathbb {R}^{d+1}_{+}$ so that

$$\biggl( \int_{\Gamma(x)} \bigl\vert f(y,t) \bigr\vert ^{q} \frac{dy\,dt}{t^{d+1}} \biggr)^{1/q}\in L^{p}\bigl(\mathbb {R}^{d}\bigr), \quad \text{when } 1\leq q< \infty, $$

and

$$\sup_{(y,t)\in\Gamma(x)} \bigl\vert f(y,t) \bigr\vert \in L^{p}\bigl(\mathbb {R}^{d}\bigr),\quad \text{when } q=\infty, $$

where $\Gamma(x)$ is the standard cone whose vertex is $x\in\mathbb {R}^{d}$, i.e.,

$$\Gamma(x)=\bigl\{ (y,t):|y-x|< t\bigr\} . $$

Assume that $B(x_{0},r)$ is a ball in $\mathbb {R}^{d}$, its tent *B̂* is defined by $\widehat{B}=\{(x,t):|x-x_{0}|\leq r-t\}$. A function $a(x,t)$ that is supported in a tent *B̂*, *B* is a ball in $\mathbb {R}^{d}$, is said to be an atom in the tent space $T^{p}_{2}$ if it satisfies

$$\biggl( \int_{\widehat{B}} \bigl|a(x,t) \bigr|^{2}\frac{dx\,dt}{t} \biggr)^{1/2}\leq |B|^{1/2-1/p}. $$

The atomic decomposition of $T^{p}_{2}$ is stated as follows.

### Proposition 2

*When* $0< p\leq1$, *then every* $f\in T^{p}_{2}$ *can be written as* $f=\sum\lambda_{k}a_{k}$, *where* $a_{k}$ *are atoms and* $\sum|\lambda_{k}|^{p}\leq C\|f\|_{T^{p}_{2}}^{p}$.

Let

$$T_{2}^{p,\infty}= \bigl\{ f(x,t): \text{measurable on } \mathbb {R}_{+}^{d+1} \text{ and } \|f\|_{T_{2}^{p,\infty}}< \infty \bigr\} , $$

where

$$\|f\|_{T_{2}^{p,\infty}}=\sup_{B\subset\mathbb{R}^{d}}\frac {1}{|B|^{1/p-1/2}} \biggl( \int_{\widehat{B}} \bigl\vert f(x,t) \bigr\vert ^{2} \frac{dx\,dt}{t} \biggr)^{1/2}. $$

Assume $0< p\leq1$, we say a function $f\in T_{2}^{p,\infty}$ belongs to the space $T_{2, 0}^{p,\infty}$ if *f* satisfies $\eta_{1}(f)=\eta_{1}2(f)=\eta_{3}(f)=0$, where

$$\begin{aligned}& \eta_{1}(f)=\lim_{r\rightarrow0}\sup_{B\subset\mathbb{R}^{d} r_{B}< r} \frac{1}{|B|^{1/p-1/2}} \biggl( \int_{\widehat{B}} \bigl\vert f(x,t) \bigr\vert ^{2} \frac{dx\,dt}{t} \biggr)^{1/2}; \\& \eta_{2}(f)=\lim_{r\rightarrow\infty}\sup_{B\subset\mathbb{R}^{d} r_{B}\geq r} \frac{1}{|B|^{1/p-1/2}} \biggl( \int_{\widehat{B}} \bigl\vert f(x,t) \bigr\vert ^{2} \frac{dx\,dt}{t} \biggr)^{1/2}; \\& \eta_{3}(f)=\lim_{r\rightarrow\infty}\sup_{B\subset B(0,r)^{c}} \frac{1}{|B|^{1/p-1/2}} \biggl( \int_{\widehat{B}} \bigl\vert f(x,t) \bigr\vert ^{2} \frac{dx\,dt}{t} \biggr)^{1/2}. \end{aligned}$$

Let $\widetilde{T_{2}^{p}}=\{F(x,t)=\sum_{i}\lambda_{i}a_{i}(x,t): a_{i}(x,t) \text{are } T_{2}^{p} \text{ atoms and } \sum_{i}|\lambda_{i}|^{p}<\infty\}$ and $\|F\|_{\widetilde{T_{2}^{p}}}=\inf\{ (\sum_{i}|\lambda_{i}|^{p} )^{1/p}: F(x,t)=\sum_{i}\lambda_{i}a_{i}(x,t)\}$. Then $\widetilde{T_{2}^{p}}$ is a Banach space. In fact, it is the completeness of $T_{2}^{p}$. Especially, $\widetilde{T_{2}^{1}}=T_{2}^{1}$.

In [19], the author proved the following result.

### Proposition 3

*Let* $0< p\leq1$. *Then*

$$\bigl(T_{2, 0}^{p,\infty} \bigr)^{*}=\widetilde{T_{2}^{p}}, \qquad \bigl(T_{2}^{p} \bigr)^{*}=T_{2}^{p,\infty}. $$

Let $\{P_{t}^{L}\}_{t>0}$ be the semigroup of linear operators generated by $-\sqrt{L}$ and $D_{t}^{L}f(x)=t|\nabla P_{t}^{L}f |(x)$, where $\nabla=(\partial_{t},\partial_{x_{1}},\ldots,\partial _{x_{d}})$. The Carleson measure characterization of the Campanato space $\Lambda_{d(1/p-1)}^{L}$ as (cf. [6]).

### Proposition 4

*Let* $\frac{d}{d+1}< p\leq1$. *Then*, *for any* $f\in L_{\mathrm{loc}}^{2}(\mathbb{R}^{d})$, *we have*:

- *If* $f \in\Lambda_{d(1/p-1)}^{L}$, *then* $D^{L}_{t}f\in T_{2}^{p,\infty}$; *moreover*, *we have*
  $$ \bigl\Vert D^{L}_{t}f \bigr\Vert _{T_{2}^{p,\infty}} \leq C \Vert f \Vert _{\Lambda_{d(1/p-1)}^{L}}. $$
- *Conversely*, *if* $f\in L^{1}((1+|x|)^{-(d+1)}\,dx)$ *and* $D^{L}_{t}f\in T_{2}^{p,\infty}$, *then* $f \in\Lambda_{d(1/p-1)}^{L}$ *and*
  $$ \Vert f \Vert _{\Lambda_{d(1/p-1)}^{L}} \leq C \bigl\Vert D^{L}_{t}f \bigr\Vert _{T_{2}^{p,\infty}}. $$

The predual space of the classical Hardy space has been studied in [19] and [16].

### Definition 2

Let $\alpha>0$, we will say a function *f* of $\Lambda_{\alpha}^{L}$ is in $\lambda_{\alpha}^{L}$ if it satisfies $\gamma_{1}(f)=\gamma_{2}(f)=\gamma_{3}(f)=0$, where

$$\begin{aligned}& \gamma_{1}(f)=\lim_{r\rightarrow0}\sup_{B\subset\mathbb{R}^{d} r_{B}< r} \frac{1}{|B|^{\alpha/d+1/2}} \biggl( \int_{\widehat{B}} \bigl\vert f-f(B, V) \bigr\vert ^{2} \frac{dx\,dt}{t} \biggr)^{1/2}; \\& \gamma_{2}(f)=\lim_{r\rightarrow\infty}\sup_{B\subset\mathbb{R}^{d} r_{B}\geq r} \frac{1}{|B|^{\alpha/d+1/2}} \biggl( \int_{\widehat{B}} \bigl\vert f-f(B, V) \bigr\vert ^{2} \frac{dx\,dt}{t} \biggr)^{1/2}; \\& \gamma_{3}(f)=\lim_{r\rightarrow\infty}\sup_{B\subset B(0,r)^{c}} \frac{1}{|B|^{\alpha/d+1/2}} \biggl( \int_{\widehat{B}} \bigl\vert f-f(B, V) \bigr\vert ^{2} \frac{dx\,dt}{t} \biggr)^{1/2}. \end{aligned}$$

The dual space of $\lambda_{d(1/p-1)}^{L}$ is $B_{L}^{p}(\mathbb{R}^{d})$, which is the completeness of $H_{L}^{p}(\mathbb{R}^{d})$ (cf. [14]).

We can give a Carleson measure characterization of $\lambda_{d(1/p-1)}^{L}$ as follows (see [14]).

### Proposition 5

*Let* $\frac{d}{d+1}< p\leq1$. *Then*, *for any* $f\in L_{\mathrm{loc}}^{2}(\mathbb{R}^{d})$, *we have*:

- *If* $f \in\lambda_{d(1/p-1)}^{L}$, *then* $D^{L}_{t}f\in T_{2}^{p,\infty}$; *moreover*, *we have*
  $$ \bigl\Vert D^{L}_{t}f \bigr\Vert _{T_{2}^{p,\infty}} \leq C \Vert f \Vert _{\lambda_{d(1/p-1)}^{L}}. $$
- *Conversely*, *if* $f\in L^{1}((1+|x|)^{-(d+1)}\,dx)$ *and* $D^{L}_{t}f\in T_{2}^{p,\infty}$, *then* $f \in\lambda_{d(1/p-1)}^{L}$ *and*
  $$ \Vert f \Vert _{\lambda_{d(1/p-1)}^{L}} \leq C \bigl\Vert D^{L}_{t}f \bigr\Vert _{T_{2}^{p,\infty}}. $$

Let $A_{j}=\partial_{x_{j}}+x_{j}$ and $A_{-j}=\partial_{x_{j}}-x_{j}$ for $j=1,2,\ldots, d$. Then

$$L= \sum_{j=1}^{d} A_{j}A_{-j}+A_{-j}A_{j}. $$

Therefore, in the harmonic analysis associated with *L*, the operators $A_{j}$ play the role of the classical partial derivatives $\partial _{x_{j}}$ in the Euclidean harmonic analysis (see [2, 11, 18]). Now, it is natural to consider the derivatives $A_{i}$ other than $\partial_{x_{j}}$. In [13], the author defined the Lusin area integral operator by $A_{j}$ and characterized the Hardy space $H_{L}^{1}(\mathbb {R}^{d})$. As a continuous study of the function spaces associated with *L*, in this paper we will define the Carleson measure by $A_{j}$ and characterize the dual spaces and predual spaces of $H_{L}^{p}(\mathbb {R}^{d})$. Moreover, let $Q_{t}^{L}f(x)=t|\tilde{\nabla} P_{t}^{L}f |(x)$, where $\tilde {\nabla}=(\partial_{t},A_{-1},\ldots,A_{-d}, A_{1},\ldots,A_{d})$. Then the main results of this paper can be stated as follows.

### Theorem 1

*Let* $\frac{d}{d+1}< p\leq1$. *Then*, *for every* $f\in L_{\mathrm{loc}}^{2}(\mathbb{R}^{d})$, *we have*:

- *If* $f \in\Lambda_{d(1/p-1)}^{L}$, *then* $Q^{L}_{t}f\in T_{2}^{p,\infty}$; *moreover*, *we have*
  $$ \bigl\Vert Q^{L}_{t}f \bigr\Vert _{T_{2}^{p,\infty}} \leq C \Vert f \Vert _{\Lambda_{d(1/p-1)}^{L}}. $$
- *Conversely*, *if* $f\in L^{1}((1+|x|)^{-(d+1)}\,dx)$ *and* $Q^{L}_{t}f\in T_{2}^{p,\infty}$, *then* $f \in\Lambda_{d(1/p-1)}^{L}$ *and*
  $$ \Vert f \Vert _{\Lambda_{d(1/p-1)}^{L}} \leq C \bigl\Vert Q^{L}_{t}f \bigr\Vert _{T_{2}^{p,\infty}}. $$

### Remark 2

In [8], the authors characterize the case $p=0$, i.e., $\mathit{BMO}_{L}$, by the heat semigroup with the classical derivatives. In [15], the authors characterize the space $\mathit{BMO}_{L}$ by the Poisson semigroup with the classical derivatives. In this paper, we will use the new derivatives $A_{j}$ of the Poisson semigroup to characterize the space $\Lambda_{d(1/p-1)}^{L}$ for $\frac{d}{d+1}< p\leq1$.

### Theorem 2

*Let* $\frac{d}{d+1}< p\leq1$. *Then*, *for any* $f\in L_{\mathrm{loc}}^{2}(\mathbb{R}^{d})$, *we have*:

- *If* $f \in\lambda_{d(1/p-1)}^{L}$, *then* $Q^{L}_{t}f\in T_{2, 0}^{p,\infty}$; *moreover*, *we have*
  $$ \bigl\Vert Q^{L}_{t}f \bigr\Vert _{T_{2, 0}^{p,\infty}} \leq C \Vert f \Vert _{\lambda_{d(1/p-1)}^{L}}. $$
- *Conversely*, *if* $f\in L^{1}((1+|x|)^{-(d+1)}\,dx)$ *and* $Q^{L}_{t}f\in T_{2, 0}^{p,\infty}$, *then* $f \in\lambda_{d(1/p-1)}^{L}$ *and*
  $$ \Vert f \Vert _{\lambda_{d(1/p-1)}^{L}} \leq C \bigl\Vert Q^{L}_{t}f \bigr\Vert _{T_{2, 0}^{p,\infty}}. $$

The paper is organized as follows. In Sect. 2, we give some estimates of the kernels. In Sect. 3, we give the proof of Theorem 1. The proofs of Theorem 2 will be given in Sect. 4.

Throughout the article, we will use *A* and *C* to denote the positive constants, which are independent of the main parameters and may be different at each occurrence. By $B_{1} \sim B_{2}$, we mean that there exists a constant $C>1$ such that $\frac{1}{C} \leq \frac{B_{1}}{B_{2}}\leq C$.

## Estimates of the kernels

In this section, we give some estimates of the kernels, which we will use in the sequel.

The proofs of these estimates can be found in [9].

### Lemma 1

- *For every* $N\in\mathbb{N}$, *there is a constant* $C_{N}>0$ *such that*
  2 $$ 0\leq K_{t}^{L}(x,y)\leq C_{N}t^{-\frac{d}{2}}e^{-(5t)^{-1}|x-y|^{2}} \biggl(1+\frac{\sqrt{t}}{\rho(x)}+\frac{\sqrt{t}}{\rho(y)} \biggr)^{-N}. $$
- *There exists* $C>0$ *such that*, *for every* $N>0$, *there is a constant* $C_{N}>0$ *so that*, *for all* $|h|\leq \sqrt{t}$,
  3 $$ \bigl\vert K_{t}^{L}(x+h,y)-K_{t}^{L}(x,y) \bigr\vert \leq C_{N}\frac{|h|}{\sqrt{t}}t^{-\frac{d}{2}} e^{-At^{-1}|x-y|^{2}} \biggl(1+\frac{\sqrt{t}}{\rho(x)}+\frac{\sqrt {t}}{\rho(y)} \biggr)^{-N}. $$

By subordination formula, we can give the following estimates about the Poisson kernel.

### Lemma 2

- *For every* *N*, *there is a constant* $C_{N}>0$, $A>0$ *such that*
  4 $$ 0\leq P_{t}^{L}(x,y)\leq C_{N} \frac{t}{(t^{2}+A|x-y|^{2})^{(d+1)/2}} \biggl(1+\frac{t}{\rho(x)}+\frac{t}{\rho(y)} \biggr)^{-N}. $$
- *Let* $|h|<\frac{|x-y|}{2}$. *Then*, *for any* $N>0$, *there exist constants* $C>0$, $C_{N}>0$ *such that*
  5 $$ \bigl\vert P_{t}^{L}(x+h,y)-P_{t}^{L}(x,y) \bigr\vert \leq C_{N}\frac{|h|}{\sqrt{t}}\frac{t}{(t^{2}+A|x-y|^{2})^{(d+1)/2}} \biggl(1+ \frac{t}{\rho(x)}+\frac{t}{\rho(y)} \biggr)^{-N}. $$

Duong et al. [6] proved the following estimates about the kernel $D_{t}^{L}(x,y)$.

### Lemma 3

*There exist constants* *C* *such that*, *for every* *N*, *there is a constant* $C_{N}>0$, *so that*

$$\begin{aligned} (\mathrm{a})&\quad \bigl\vert D_{t}^{L}(x,y) \bigr\vert \leq C_{N}t^{-d} e^{-Ct^{-2} \vert x-y \vert ^{2}}\biggl(1+ \frac{t}{\rho(x)}+\frac{t}{\rho (y)}\biggr)^{-N} ; \\ (\mathrm{b})&\quad \bigl\vert D_{t}^{L}(x+h,y)-D_{t}^{L}(x,y) \bigr\vert \leq C_{k,N}\biggl(\frac{ \vert h \vert }{t}\biggr)^{\delta'}t^{-d} e^{-Ct^{-2} \vert x-y \vert ^{2}}\biggl(1+\frac{t}{\rho(x)}+\frac{t}{\rho (y)} \biggr)^{-N}, \\ &\qquad \textit{for all } \vert h \vert \leq t; \\ (\mathrm{c})&\quad \biggl\vert \int_{\mathbb {R}^{d}}D_{t}^{L}(x,y)\,dy \biggr\vert \leq C_{N}\frac{t/\rho(x)}{(1+t/\rho(x))^{N}}. \end{aligned}$$

Let $t=\frac{1}{2}\ln\frac{1+s}{1-s}$, $s\in(0,1)$. Then

6 $$ K_{t}^{L}(x,y)= \biggl(\frac{1-s^{2}}{4\pi s} \biggr)^{d/2}\exp{ \biggl(-\frac{1}{4}\biggl(s|x+y|^{2}+ \frac{1}{s}|x-y|^{2}\biggr) \biggr)}\doteq K_{s}(x,y). $$

The following estimations are very important for the proofs of the main result in this paper.

### Lemma 4

*There is* $C>0$ *for* $N\in\mathbb {N}$ *and* $|x-x'|\leq\frac{|x-y|}{2}$, *any* $j=-1,\ldots,-d,1,\ldots,d$, *we can find* $C_{N}>0$ *such that*

$$\begin{aligned} (\mathrm{a})&\quad \bigl\vert \sqrt{t}A_{j}K_{t}^{L}(x,y) \bigr\vert \leq C_{N}t^{-\frac {d}{2}}\exp{ \biggl(- \frac{ \vert x-y \vert ^{2}}{C t} \biggr)}\biggl(1+\frac{\sqrt {t}}{\rho(x)}+\frac{\sqrt{t}}{\rho(y)} \biggr)^{-N} ; \\ (\mathrm{b})&\quad \bigl\vert \sqrt{t}A_{j}K_{t}^{L}(x,y)- \sqrt{t}A_{j}K_{t}^{L}\bigl(x',y \bigr) \bigr\vert \\ &\qquad \leq C_{N}\frac{ \vert x-x' \vert }{t}t^{-\frac{d}{2}}\exp{ \biggl(- \frac{ \vert x-y \vert ^{2}}{C t} \biggr)}\biggl(1+\frac{\sqrt{t}}{\rho(x)}+\frac{\sqrt{t}}{\rho (y)} \biggr)^{-N}; \\ (\mathrm{c})&\quad \biggl\vert \int_{\mathbb {R}^{d}}\sqrt{t}A_{j}K_{t}^{L}(x,y) \,dy \biggr\vert \leq C\frac{t/\rho(x)}{(1+t/\rho(x))^{N}}. \end{aligned}$$

### Proof

By

$$\begin{aligned} \bigl\vert A_{j}K_{t}^{L}(x,y) \bigr\vert =& \biggl\vert \frac{\partial}{\partial x_{j}}K_{t}^{L}(x,y)+x_{j}K_{t}^{L}(x,y) \biggr\vert \\ \leq& \biggl\vert \frac{\partial}{\partial x_{j}}K_{t}^{L}(x,y) \biggr\vert + \bigl\vert x_{j}K_{t}^{L}(x,y) \bigr\vert \doteq I_{1}+I_{2}, \end{aligned}$$

and $t=\frac{1}{2}\ln\frac{1+s}{1-s}\sim s$, $s\rightarrow0^{+}$, for $s\in(0,\frac{1}{2}]$, we have

$$\begin{aligned} I_{2} \le&C|x_{j}|s^{-\frac{d}{2}}\exp{ \biggl(- \frac {1}{4}s|x+y|^{2} \biggr)}\exp{ \biggl(-\frac{1}{4} \frac {|x-y|^{2}}{s} \biggr)} \\ \leq&C|x|s^{-\frac{d}{2}}\exp{ \biggl(-\frac{1}{4}s|x+y|^{2} \biggr)}\exp{ \biggl(-\frac{1}{4}\frac{|x-y|^{2}}{s} \biggr)}. \end{aligned}$$

If $x\cdot y\leq0$, then $|x|\leq|x-y|$. So

$$\begin{aligned} I_{2} \le&Cs^{-\frac{d}{2}}|x-y|\exp{ \biggl(-\frac{1}{4} \frac {|x-y|^{2}}{s} \biggr)}\leq C s^{-\frac{d-1}{2}}\exp{ \biggl(-\frac {|x-y|^{2}}{8s} \biggr)} \\ \leq&C t^{-\frac{d-1}{2}}\exp{ \biggl(-\frac{|x-y|^{2}}{8t} \biggr)}. \end{aligned}$$

If $x\cdot y\geq0$, then $|x|\leq|x+y|$. So

$$\begin{aligned} I_{2} \le&Cs^{-\frac{d}{2}}|x+y|\exp{ \biggl(-\frac {1}{4}s|x+y|^{2} \biggr)}\exp{ \biggl(-\frac{1}{4}\frac {|x-y|^{2}}{s} \biggr)} \\ \leq& C s^{-\frac{d+1}{2}}\exp{ \biggl(-\frac{|x-y|^{2}}{4s} \biggr)} \leq C t^{-\frac{d+1}{2}}\exp{ \biggl(-\frac{|x-y|^{2}}{4t} \biggr)}. \end{aligned}$$

Therefore,

7 $$ \vert \sqrt{t}I_{2} \vert \leq C(1+t)t^{-\frac{d}{2}} \exp{ \biggl(-\frac {|x-y|^{2}}{8t} \biggr)}\leq Ct^{-\frac{d}{2}}\exp{ \biggl(- \frac {|x-y|^{2}}{8t} \biggr)}. $$

Since

$$\lim_{t\rightarrow\infty}t^{2} \biggl(1- \biggl(\frac {e^{2t}-1}{e^{2t}+1} \biggr)^{2} \biggr)=0, $$

we get $(\frac{1-s^{2}}{4\pi s} )^{d/2}\leq t^{-d}$ for $s\in[\frac{1}{2}, 1)$.

When $s\in[\frac{1}{2},1)$, we get $t=\frac{1}{2}\ln\frac {1+s}{1-s}> s$. Therefore

$$\begin{aligned} I_{2} \leq&C|x_{j}|\exp{ \biggl(-\frac{1}{4} \biggl(s|x+y|^{2}+\frac {|x-y|^{2}}{s}\biggr) \biggr)} \\ \leq&Ct^{-d}|x|\exp{ \biggl(-\frac{1}{4}\biggl(s|x+y|^{2}+ \frac {|x-y|^{2}}{s}\biggr) \biggr)} \\ \leq&Ct^{-d}\bigl( \vert x+y \vert + \vert x-y \vert \bigr) \exp{ \biggl(-\frac{1}{4}\biggl(s|x+y|^{2}+\frac {|x-y|^{2}}{s} \biggr) \biggr)} \\ \leq&Ct^{-d}\exp{ \biggl(-\frac{|x-y|^{2}}{8s} \biggr)} \\ \leq& Ct^{-d}\exp{ \biggl(-\frac{|x-y|^{2}}{8t} \biggr)}. \end{aligned}$$

Then

8 $$ \vert \sqrt{t}I_{2} \vert \leq Ct^{-d+\frac{1}{2}} \exp{ \biggl(-\frac {|x-y|^{2}}{8t} \biggr)}\leq Ct^{-\frac{d}{2}}\exp{ \biggl(- \frac {|x-y|^{2}}{8t} \biggr)}. $$

By (6), we get

$$\frac{\partial}{\partial x_{j}}K_{s}(x,y)=-\frac{1}{2}\biggl(s(x_{j}+y_{j})+ \frac {1}{s}(x_{j}-y_{j})\biggr)K_{s}(x,y) $$

and

$$ I_{1}\leq C\biggl(s|x_{j}+y_{j}|+ \frac{1}{s}|x_{j}-y_{j}|\biggr)K_{s}(x,y) \leq C\biggl(s|x+y|+\frac {1}{s}|x-y|\biggr)K_{s}(x,y). $$

Therefore, when $s\in(0,\frac{1}{2}]$, we have

$$I_{1}\leq C s^{-\frac{d}{2}}(1+s)\exp{ \biggl(-\frac{|x-y|^{2}}{8s} \biggr)}\leq Ct^{-\frac{d}{2}}\exp{ \biggl(-\frac{|x-y|^{2}}{8t} \biggr)}. $$

When $s\in[\frac{1}{2},1)$, we have

$$I_{1}\leq C t^{-d}\exp{ \biggl(-\frac{|x-y|^{2}}{8s} \biggr)} \leq Ct^{-d}\exp{ \biggl(-\frac{|x-y|^{2}}{8t} \biggr)}. $$

Then

9 $$ \biggl\vert \sqrt{t}\frac{\partial}{\partial x_{j}}K_{t}^{L}(x,y) \biggr\vert \leq Ct^{-\frac{d}{2}}\exp{ \biggl(-\frac{|x-y|^{2}}{8t} \biggr)}. $$

By (7)–(9), we get

10 $$ \bigl\vert \sqrt{t}A_{j}K_{t}^{L}(x,y) \bigr\vert \leq Ct^{-\frac{d}{2}}\exp{ \biggl(-\frac{|x-y|^{2}}{8t} \biggr)}. $$

Similar to the proof of (10), for any $N>0$, we can prove

$$\bigl(\sqrt{t} \vert x \vert \bigr)^{N} \bigl\vert \sqrt{t}A_{j}K_{t}^{L}(x,y) \bigr\vert \leq C_{N}t^{-\frac{d}{2}}\exp { \biggl(-\frac{ \vert x-y \vert ^{2}}{8t} \biggr)} $$

and

$$t^{N} \bigl\vert \sqrt{t}A_{j}K_{t}^{L}(x,y) \bigr\vert \leq C_{N}t^{-\frac{d}{2}}\exp{ \biggl(-\frac { \vert x-y \vert ^{2}}{8t} \biggr)}. $$

Since $\rho(x)=\frac{1}{1+|x|}$, we get $\frac{\sqrt{t}}{\rho(x)}=\sqrt {t}(1+|x|)$. Then, for $N>0$,

11 $$ \biggl(\frac{\sqrt{t}}{\rho(x)} \biggr)^{N} \bigl\vert \sqrt {t}A_{j}K_{t}^{L}(x,y) \bigr\vert \leq C_{N}t^{-\frac{d}{2}}\exp{ \biggl(-\frac { \vert x-y \vert ^{2}}{8t} \biggr)}. $$

Since *x* and *y* are symmetric, we also have

12 $$ \biggl(\frac{\sqrt{t}}{\rho(y)} \biggr)^{N} \bigl\vert tA_{j}K_{t}^{L}(x,y) \bigr\vert \leq C_{N}t^{-\frac{d}{2}}\exp{ \biggl(-\frac{ \vert x-y \vert ^{2}}{8t} \biggr)}. $$

Then (a) follows from (10)–(12).

(b) Note that

$$\begin{aligned}& \bigl\vert \sqrt{t}A_{j}K_{t}^{L} \bigl(x',y\bigr)-\sqrt{t}A_{j}K_{t}^{L}(x,y) \bigr\vert \\& \quad \leq \biggl\vert \sqrt{t}\frac{\partial}{\partial x_{j}}K_{t}^{L} \bigl(x',y\bigr)-\sqrt {t}\frac{\partial}{\partial x_{j}}K_{t}^{L}(x,y) \biggr\vert + \bigl\vert \sqrt{t}x'_{j}K_{t}^{L} \bigl(x',y\bigr)-\sqrt{t}x_{j}K_{t}^{L}(x,y) \bigr\vert \\& \quad \doteq J_{1}+J_{2}. \end{aligned}$$

For $J_{2}$, let

$$ \varphi(z)=\varphi_{y,s}(z)=z_{j}\exp{ \biggl(- \frac{1}{4}\alpha (s,z,y) \biggr)}, $$

where $\alpha(s,z,y)=s|z+y|^{2}+\frac{1}{s}|z-y|^{2}$.

Then

$$\frac{\partial\varphi}{\partial z_{k}}(z)= \biggl(\delta_{jk}-\frac {s}{2}z_{j}(z_{k}+y_{k})- \frac{1}{2s}z_{j}(z_{k}-y_{k}) \biggr)\exp{ \biggl(-\frac {1}{4}\alpha(s,z,y) \biggr)}. $$

Therefore

13 $$\begin{aligned} \biggl\vert \frac{\partial\varphi}{\partial z_{k}}(z) \biggr\vert \leq &C\biggl(1+s \vert z \vert \vert z+y \vert +\frac{1}{s} \vert z \vert \vert z-y \vert \biggr)\exp{ \biggl(-\frac{1}{4}\alpha (s,z,y) \biggr)} \\ \leq&C\biggl(1+s^{1/2} \vert z \vert +\frac{1}{s^{1/2}} \vert z \vert \biggr)\exp{ \biggl(-\frac {1}{8}\alpha(s,z,y) \biggr)} \\ \leq&C\biggl(1+s^{1/2}\bigl( \vert z-y \vert + \vert z+y \vert \bigr)+\frac{1}{s^{1/2}}\bigl( \vert z-y \vert + \vert z+y \vert \bigr) \biggr)\exp { \biggl(-\frac{1}{8}\alpha(s,z,y) \biggr)} \\ \leq&C\biggl(1+s+\frac{1}{s}\biggr)\exp{ \biggl(-\frac{1}{16s} \vert z-y \vert ^{2} \biggr)} \\ \leq&Cs^{-1}\exp{ \biggl(-\frac{1}{16s} \vert z-y \vert ^{2} \biggr)}. \end{aligned}$$

Let $\theta=\lambda x+(1-\lambda)x'$, $0<\lambda<1$. Then

$$\begin{aligned} J_{2} \leq&Ct^{-d/2} \bigl\vert x'_{j}K_{s} \bigl(x',y\bigr)-x_{j}K_{s}(x,y) \bigr\vert \\ \leq&Ct^{-d/2} \bigl\vert x-x' \bigr\vert \sup _{\theta} \bigl\vert \nabla\varphi(\theta) \bigr\vert \\ \leq&Ct^{-d/2}\frac{|x-x'|}{s}|\sup_{\theta}\exp{ \biggl(-\frac {|\theta-y|^{2}}{16s} \biggr)} \\ \leq&Ct^{-d/2}\frac{|x-x'|}{t}|\sup_{\theta}\exp{ \biggl(-\frac {|\theta-y|^{2}}{16t} \biggr)}. \end{aligned}$$

When $|x-x'|\leq\frac{|x-y|}{2}$, we can get $|\theta-y|\sim|x-y|$. Therefore, there exists $A>0$ such that

14 $$ J_{2}\leq Ct^{-d/2}\frac{|x-x'|}{t}\exp{ \biggl(-\frac {|x-y|^{2}}{At} \biggr)}. $$

For $J_{1}$,

$$\begin{aligned} J_{1} =& \biggl\vert \sqrt{t}\frac{\partial}{\partial x_{j}}K_{t}^{L} \bigl(x',y\bigr)-\sqrt {t}\frac{\partial}{\partial x_{j}}K_{t}^{L}(x,y) \biggr\vert \\ =&\sqrt{t} \biggl\vert \frac{\partial}{\partial x_{j}}K_{s} \bigl(x',y\bigr)-\frac {\partial}{\partial x_{j}}K_{s}(x,y) \biggr\vert \\ =&\sqrt{t} \biggl\vert \biggl(s(x_{j}+y_{j})+ \frac{1}{s}(x_{j}-y_{j})\biggr)\exp{ \biggl(- \frac{1}{4}\alpha(s,x,y) \biggr)} \\ &{}-\biggl(s\bigl(x'_{j}+y_{j}\bigr)+ \frac{1}{s}\bigl(x'_{j}-y_{j}\bigr) \biggr)\exp{ \biggl(-\frac{1}{4}\alpha \bigl(s,x',y\bigr) \biggr)} \biggr\vert . \end{aligned}$$

Let

$$\psi(z)=\psi_{y,s}(z)=\biggl(s(z_{j}+y_{j})+ \frac{1}{s}(z_{j}-y_{j})\biggr)\exp{ \biggl(- \frac{1}{4}\alpha(s,z,y) \biggr)}. $$

Then

$$\begin{aligned} \frac{\partial\psi}{\partial z_{k}}(z) =&\biggl[\biggl(s+\frac{1}{s}\biggr)\delta _{jk}-\frac{1}{2}\biggl(s(z_{j}+y_{j})+ \frac{1}{s}(z_{j}-y_{j})\biggr) \\ &{}s(z_{k}+y_{k})+\frac{1}{s}(z_{k}-y_{k}) \biggr]\exp{ \biggl(-\frac{1}{4}\alpha (s,z,y) \biggr)}. \end{aligned}$$

Therefore, similar to the proofs of (13) and (14), we can prove

$$\biggl\vert \frac{\partial\psi}{\partial z_{k}}(z) \biggr\vert \leq Cs^{-1}\exp { \biggl(-\frac{1}{4}\alpha(s,z,y) \biggr)} $$

and

15 $$\begin{aligned} J_{1} \leq&C\sup_{\theta}\bigl\vert \nabla\psi( \theta) \bigr\vert \bigl\vert x-x' \bigr\vert \\ \leq&Ct^{-d/2}\frac{|x-x'|}{t}|\exp{ \biggl(-\frac {|x-y|^{2}}{At} \biggr)}. \end{aligned}$$

Inequalities (13) and (15) show

$$ \bigl\vert \sqrt{t}A_{j}K_{t}^{L}(x,y)- \sqrt{t}A_{j}K_{t}^{L}\bigl(x',y \bigr) \bigr\vert \leq C_{N}\frac{|x-x'|}{t}t^{-\frac{d}{2}}\exp{ \biggl(-\frac {|x-y|^{2}}{At} \biggr)}. $$

Then, similar to the proof of (a), we have

$$\bigl\vert \sqrt{t}A_{j}K_{t}^{L}(x,y)- \sqrt{t}A_{j}K_{t}^{L}\bigl(x',y \bigr) \bigr\vert \leq C_{N}\frac{|x-x'|}{t}t^{-\frac{d}{2}}\exp{ \biggl(-\frac {|x-y|^{2}}{At} \biggr)} \biggl(1+\frac{\sqrt{t}}{\rho(x)}+\frac{\sqrt {t}}{\rho(y)} \biggr)^{-N}. $$

(c) Noting that

$$\begin{aligned}& \biggl\vert \int_{\mathbb {R}^{d}}\sqrt{t}A_{j}K_{t}^{L}(x,y) \,dy \biggr\vert \\& \quad \leq \biggl\vert \int_{\mathbb {R}^{d}}\sqrt{t}\partial _{x_{j}}K_{t}^{L}(x,y) \,dy \biggr\vert + \biggl\vert \int_{\mathbb {R}^{d}}\sqrt {t}x_{j}K_{t}^{L}(x,y) \,dy \biggr\vert \\& \quad \doteq I+\mathit{II}. \end{aligned}$$

The proof of part *I* can be found in Lemma 3.9 of [6]. For part *II*, since $|x_{j}|\leq1+|x|=\frac{1}{\rho(x)}$ and Lemma 1, we get

$$\mathit{II}\leq\frac{\sqrt{t}}{\rho(x)} \int_{\mathbb {R}^{d}}\sqrt {t} \bigl\vert K_{t}^{L}(x,y) \bigr\vert \,dy\leq\frac{\frac{\sqrt{t}}{\rho(x)}}{ (1+\frac{\sqrt{t}}{\rho(x)} )^{N}}. $$

Therefore, part (c) holds and this completes the proof of Proposition 4. □

Lemma 4 and the subordination formula give the following.

### Lemma 5

*There is* $C>0$ *for* $N\in\mathbb {N}$ *and* $|x-x'|\leq\frac{|x-y|}{2}$, *we can find* $C_{N}>0$ *such that*

$$\begin{aligned} (\mathrm{a})&\quad \bigl\vert Q_{t}^{L}(x,y) \bigr\vert \leq C_{N}\frac{t}{(t^{2}+A \vert x-y \vert ^{2})^{(d+1)/2}} \biggl(1+\frac{t}{\rho(x)}+ \frac{t}{\rho(y)}\biggr)^{-N}; \\ (\mathrm{b})&\quad \bigl\vert Q_{t}^{L}(x,y)-Q_{t}^{L} \bigl(x',y\bigr) \bigr\vert \leq C_{N} \biggl( \frac{ \vert x-x' \vert }{t} \biggr)\frac{t}{(t^{2}+A \vert x-y \vert ^{2})^{(d+1)/2}} \biggl(1+\frac{t}{\rho(x)}+ \frac{t}{\rho(y)}\biggr)^{-N}; \\ (\mathrm{c})&\quad \biggl\vert \int_{\mathbb {R}^{d}}Q_{t}^{L}(x,y)\,dy \biggr\vert \leq C_{N}\frac{t/\rho(x)}{(1+t/\rho(x))^{N}}. \end{aligned}$$

## Carleson measure characterization of $\Lambda_{\alpha}^{L}$

Let $s_{L}$ denote the Littlewood–Paley *g*-function associated with *L*, i.e.,

$$s_{L}f(x)= \biggl( \int_{0}^{\infty} \bigl\vert Q_{t}^{L}f(x) \bigr\vert ^{2}\frac {dt}{t} \biggr)^{1/2}, $$

and $A_{L}$ denote the Lusin area integral associated with *L*, i.e.,

$$A_{L}f(x)= \biggl( \int_{0}^{\infty} \int_{\Gamma(x)} \bigl\vert Q_{t}^{L}f(x) \bigr\vert ^{2}\frac{dy\,dt}{t} \biggr)^{1/2}. $$

Then we can prove the following.

### Lemma 6

*The operators* $s_{L}$ *and* $A_{L}$ *are isometries on* $L^{2}(\mathbb{R}^{d})$ *up to constant factors*. *Exactly*,

$$\|s_{L}f\|_{L^{2}}=\frac{1}{2}\|f\|_{L^{2}}, \qquad \|A_{L}f\|_{L^{2}}=C_{d}\|f\|_{L^{2}}. $$

The proof of Lemma 6 is standard, we omit it.

Let $F(x,t)=Q_{t}^{L}f(x)$ and $G(x,t)=Q_{t}^{L}g(x)$. Then we have the following lemma.

### Lemma 7

*If* $g\in L^{1} ((1+|x|)^{-(d+1)}\,dx )$ *and* *f* *is an* $H_{L}^{p, \infty}$-*atom*, *then*

$$\frac{1}{4} \int_{\mathbb{R}^{d}}f(x)\overline{g(x)}\,dx= \int _{\mathbb{R}_{+}^{d+1}}F(x,t)G(x,t)\frac{dx\,dt}{t}. $$

### Lemma 8

*There exists* $C>0$ *such that*, *for any* $H_{L}^{p, \infty}$-*atom* $a(x)$, *we have* $\|A_{L}a\|_{L^{p}}\leq C$.

The proofs of Lemmas 7 and 8 can be found in [8].

Now we can give the proof of Theorem 1.

### Proof of Theorem 1

Let $f \in \Lambda_{d(1/p-1)}^{L}$, then $f\in L^{1} ((1+|x|)^{-(d+1)}\,dx )$. By Lemma 5(a), we know

$$ Q^{L}_{t} f(x)= \int_{\mathbb{R}^{d}} Q^{L}_{t}(x,y) f(y) \,dy $$

is absolutely convergent. To prove the assertion (a), we need to prove that, for any ball $B=B(x_{0},r)$,

16 $$ \frac{1}{|B|^{2/p-1}} \int_{\widehat{B}} \bigl\vert Q^{L}_{t} f(x) \bigr\vert ^{2} \frac{dx \,dt}{t} \leq C \| f \|^{2}_{\Lambda_{d(1/p-1)}^{L}}. $$

Set $B_{k}=B(x_{0},2^{k}r)$ and

$$ f = \bigl( f - f(B_{1}) \bigr) \chi_{{B_{1}}}+ \bigl( f - f(B_{1}) \bigr) \chi_{{B^{c}_{1}}}+ f(B_{1})= \widetilde{f}_{1} + \widetilde{f}_{2} + f(B_{1}). $$

By Lemma 6, we have

17 $$\begin{aligned} \frac{1}{|B|^{2/p-1}} \int_{\widehat{B}} \bigl\vert Q^{L}_{t} \widetilde{f}_{1}(x) \bigr\vert ^{2} \frac{dx \,dt}{t} \leq&\frac{1}{|B|^{2/p-1}} \int_{B} \bigl\vert s_{L} \widetilde{f}_{1}(x) \bigr\vert ^{2} \,dx \\ =& \frac{1}{4|B|^{2/p-1}} \| \widetilde{f}_{1} \|^{2}_{L^{2}} = \frac{1}{4|B|^{2/p-1}} \int_{B_{1}} \bigl\vert f(g)- f(B_{1}) \bigr\vert ^{2} \,dx \\ \leq& C \| f \|^{2}_{\Lambda_{d(1/p-1)}^{L}}. \end{aligned}$$

Note that

$$\begin{aligned} \bigl\vert f(B_{2})- f(B_{1}) \bigr\vert \leq& 2^{d}\frac{1}{ \vert B_{2} \vert } \int_{B_{2}} \bigl\vert f(x)-f(B_{2}) \bigr\vert \,dx \\ \leq&2^{d}\frac{1}{ \vert B_{2} \vert ^{1/2}} \biggl( \int_{B_{2}} \bigl\vert f(x)-f(B_{2}) \bigr\vert ^{2}\,dx \biggr)^{1/2} \\ =&2^{d} \vert B_{2} \vert ^{1/p-1} \frac{1}{ \vert B_{2} \vert ^{1/p-1/2}} \biggl( \int_{B_{2}} \bigl\vert f(x)-f(B_{2}) \bigr\vert ^{2}\,dx \biggr)^{1/2} \\ \leq&2^{d} \vert B_{2} \vert ^{1/p-1}\| f \|_{\Lambda_{d(1/p-1)}^{L}}. \end{aligned}$$

Therefore

18 $$ \bigl\vert f(B_{k+1})- f(B_{1}) \bigr\vert \leq C k |B_{k+1}|^{1/p-1} \| f \|_{\Lambda_{d(1/p-1)}^{L}}. $$

For $x \in B(x_{0},r)$, by Lemma 5(a) and (18),

$$\begin{aligned} \bigl\vert Q^{L}_{t} \widetilde{f}_{2}(x) \bigr\vert \leq& C \int_{\mathbb{R}^{d}} \frac{t}{(t^{2}+C \vert x-y \vert ^{2})^{(d+1)/2}} \bigl\vert \widetilde{f}_{2}(y) \bigr\vert \,dy \\ \leq& C \int_{(B_{1})^{c}} \frac{t}{ \vert x_{0}-y \vert ^{(d+1)}} \bigl\vert f(y)-f(B_{1}) \bigr\vert \,dy \\ \leq& C \sum^{\infty}_{k=1} \frac{t}{(2^{k} r)^{d+1}} \biggl( \int_{B_{k+1} \setminus B_{k}} \bigl\vert f(y) - f(B_{k+1}) \bigr\vert \,dy + \bigl(2^{k} r\bigr)^{d} \bigl\vert f(B_{k+1})- f(B_{1}) \bigr\vert \biggr) \\ \leq& C \frac{t}{r^{1-d(1/p-1)}} \sum^{\infty}_{k=1} 2^{k({d(1/p-1)}-1)}(1+k) \| f \|_{\Lambda_{d(1/p-1)}^{L}} \\ \leq& C \frac{t}{r^{1-d(1/p-1)}} \| f \|_{\Lambda_{d(1/p-1)}^{L}}. \end{aligned}$$

In the last step of the above, we use the facts $\frac{d}{d+1}< p\leq1$ to get $d(1/p-1)-1<0$.

Thus we have

19 $$ \frac{1}{|B|^{2/p-1}} \int_{\widehat{B}} \bigl\vert Q^{L}_{t} \widetilde{f}_{2}(x) \bigr\vert ^{2} \frac{dx \,dt}{t} \leq C \| f \|^{2}_{\Lambda_{d(1/p-1)}^{L}}. $$

It remains to estimate the constant term. Assume first that $r< \rho(x_{0})$. Taking $k_{0}$ such that $2^{k_{0}}r< \rho(x_{0}) \leq 2^{k_{0}+1}r$, we have

$$\begin{aligned} \bigl\vert f(B_{1}) \bigr\vert \leq& \bigl\vert f(B_{k_{0}+1})- f(B_{1}) \bigr\vert + \bigl\vert f(B_{k_{0}+1}) \bigr\vert \\ \leq& C k_{0} \vert B_{k_{0}+1} \vert ^{1/p-1}\| f \|_{\Lambda_{d(1/p-1)}^{L}} + \vert B_{k_{0}+1} \vert ^{1/p-1}\| f \|_{\Lambda_{d(1/p-1)}^{L}} \\ \leq& C \biggl( 1+ \log_{2} \frac{\rho(x_{0})}{r} \biggr) \vert B_{k_{0}+1} \vert ^{1/p-1}\| f \|_{\Lambda_{d(1/p-1)}^{L}} . \end{aligned}$$

Note that $\rho(x) \sim\rho(x_{0})>r$ for any $x \in B(x_{0},r)$, by using Lemma 5(c), we get

20 $$\begin{aligned}& \frac{1}{|B|^{2/p-1}} \int_{\widehat{B}} \bigl\vert Q^{L}_{t} \bigl( f(B_{1}) \mathbf{1} \bigr) (x) \bigr\vert ^{2} \frac{dx \,dt}{t} \\& \quad = \frac{ | f(B_{1}) |^{2}}{|B|^{2/p-1}} \int_{\widehat{B}} \biggl\vert \int_{\mathbb{R}^{d}} Q^{L}_{t}(x,y) \,dy \biggr\vert ^{2} \frac{dx \,dt}{t} \\& \quad \leq \frac{C | f(B_{1}) |^{2}}{|B|^{2/p-1}} \int_{\widehat{B}} \biggl( \frac{t}{\rho(x_{0})} \biggr)^{2} \frac{dx \,dt}{t} \\& \quad \leq C \frac{|B_{k_{0}+1}|^{2/p-2}}{|B|^{2/p-2}} \biggl( 1+ \log_{2}\frac{\rho(x_{0})}{r} \biggr)^{2} \biggl( \frac{r}{\rho(x_{0})} \biggr)^{2} \|f \|^{2}_{\Lambda_{d(1/p-1)}^{L}} \\& \quad = C \biggl( 1+ \log_{2} \frac{\rho(x_{0})}{r} \biggr)^{2} \biggl( \frac{r}{\rho(x_{0})} \biggr)^{2-2d(1/p-1)} \| f \|^{2}_{\Lambda_{d(1/p-1)}^{L}} \\& \quad \leq C \| f \|^{2}_{\Lambda_{d(1/p-1)}^{L}}. \end{aligned}$$

In the last step of the above, we use the fact $d(1/p-1)<1$. For $r \geq\rho(x_{0})$, we have $| f(B_{1}) | \leq C |B_{1}|^{1/p-1} \| f \|_{\Lambda_{d(1/p-1)}^{L}}$.

Note that $\rho(x) \leq Cr$ for any $x \in B(x_{0},r)$, again by Lemma 5(c), we get

21 $$\begin{aligned}& \frac{1}{|B|^{2/p-1}} \int_{\widehat{B}} \bigl\vert Q^{L}_{t} \bigl( f(B_{1})\mathbf{1} \bigr) (x) \bigr\vert ^{2} \frac{dx \,dt}{t} \\& \quad \leq\frac{ |f(B_{1}) |^{2}}{|B|^{2/p-1}} \int^{\infty}_{0} \int_{B} \biggl\vert \int_{\mathbb{R}^{d}} Q^{L}_{t}(x,y) \,dy \biggr\vert ^{2} \frac{dx \,dt}{t} \\& \quad \leq\frac{C | f(B_{1}) |^{2}}{|B|^{2/p-1}} \biggl( \int_{B} \int^{\rho(x)}_{0} \biggl( \frac{t}{\rho(x)} \biggr)^{2} \frac{dt}{t} \,dx + \int_{B} \int^{\infty}_{\rho(x)} \biggl( \frac{t}{\rho(x)} \biggr)^{-2} \frac{dt}{t} \,dx \biggr) \\& \quad \leq C \| f \|^{2}_{\Lambda_{d(1/p-1)}^{L}}. \end{aligned}$$

Then (17) follows from (18)–(21). This proves part (a).

Let $f\in L^{1} ((1+|x|)^{-(d+1)}\,dx )$ and $Q_{t}^{L}f(x)\in T_{2}^{p,\infty}$. We want to prove that $f\in \Lambda_{d(1/p-1)}^{L}$. By $\Lambda_{d(1/p-1)}^{L}$ is the dual space of $H_{L}^{p}(\mathbb {R}^{d})$, it is sufficient to prove that

$$H_{L}^{p}\ni g\mapsto\mathcal{L}_{f}(g):= \int_{\mathbb{R}^{d}}f(x)g(x)\,dx $$

defined on finite linear combinations of $H_{L}^{p,\infty}$-atoms satisfies the estimate

$$\bigl\vert \mathcal{L}_{f}(g) \bigr\vert \leq C \bigl\Vert Q_{t}^{L}f \bigr\Vert _{T_{2}^{p, \infty}}\|g \|_{H_{L}^{p}}. $$

By Lemma 7, Lemma 8, and Proposition 3, we get

$$\begin{aligned} \bigl\vert \mathcal{L}_{f}(g) \bigr\vert =& \biggl\vert \int_{\mathbb{R}^{d}}f(x)g(x)\, dx \biggr\vert \\ =&4 \biggl\vert \int_{\mathbb{R}^{d+1}_{+}}Q_{t}^{L}f(x)Q_{t}^{L}g(x) \frac{dx \,dt}{t} \biggr\vert \\ \leq&C \bigl\Vert Q_{t}^{L}f \bigr\Vert _{T_{2}^{p,\infty}} \bigl\Vert Q_{t}^{L}g \bigr\Vert _{T_{2}^{p}} \\ \leq&C \bigl\Vert Q_{t}^{L}f \bigr\Vert _{T_{2}^{p,\infty}} \Vert g \Vert _{H_{L}^{p}}. \end{aligned}$$

This gives the proof of part (b) and then Theorem 1 is proved. □

## The predual space of Hardy space $H_{L}^{p}(\mathbb{R}^{d})$

In this section, we give a Carleson measure characterization of the space $\lambda_{d(1/p-1)}^{L}(\mathbb{R}^{d})$.

### Proof of Theorem 2

Let $f\in\lambda_{d(1/p-1)}^{L}$, then $f\in \Lambda_{d(1/p-1)}^{L}$. By Theorem 1, we know $f\in L^{1} ((1+|x|)^{-(d+1)}\,dx )$. To prove $Q_{t}^{L} f\in T_{2, 0}^{p,\infty}$, we first prove that there exists a constant $C>0$ such that, for any ball $B=B(x_{0}, r)$, we have

22 $$ \frac{1}{|B|^{2/p-1}} \int_{\widehat{B}} \bigl\vert Q_{t}^{L} f(x) \bigr\vert ^{2}\frac{dx\,dt}{t}\leq \sum _{k=1}^{\infty}2^{-k(1-d(1/p-1))}\beta_{k}(f, B), $$

where

$$\beta_{k}(f,B)=\sup_{B'\subset B_{k+1}}\frac{1}{|B'|^{2/p-1}} \int_{B'} \bigl\vert f(y)-f\bigl(B'\bigr) \bigr\vert ^{2} \,dy. $$

We first assume (22) holds, then we show that $Q_{t}^{k} f\in T_{2, 0}^{p,\infty}$. In fact, as $f\in\lambda_{d(1/p-1)}^{L}$, we have $f\in \Lambda_{d(1/p-1)}^{L}$ and there exists a constant $C>0$ such that

$$\beta_{k}(f,B)\leq C \|f\|_{\Lambda_{d(1/p-1)}^{L}}. $$

Then, for any $k\in\mathbb{N}$, we have

23 $$ \lim_{a\rightarrow0}\sup_{B\subset\mathbb{R}^{d} r_{B}\leq a}\beta _{k}(f,B)=\lim_{a\rightarrow\infty}\sup_{B\subset\mathbb{R}^{d} r_{B}\geq a} \beta_{k}(f,B)=\lim_{a\rightarrow\infty}\sup_{B\subset \mathbb{R}^{d} B\subset B(0,a)^{c}} \beta_{k}(f,B)=0. $$

By (22), we have

$$\begin{aligned}& \frac{1}{|B|^{2/p-1}} \int_{\widehat{B}} \bigl\vert Q_{t}^{L} f(x) \bigr\vert ^{2}\frac{dx\,dt}{t} \\& \quad \leq C\sum_{k=1}^{k_{0}}2^{-k(1-d(1/p-1))} \beta_{k}(f,B)+C\sum_{k=k_{0}}^{\infty} 2^{-k(1-d(1/p-1))}\|f\|_{\Lambda_{d(1/p-1)}^{L}}^{2} \\& \quad \leq C\sum_{k=1}^{k_{0}}2^{-k(1-d(1/p-1))} \beta_{k}(f,B)+C2^{-k_{0}(1-d(1/p-1))} \|f\|_{\Lambda_{d(1/p-1)}^{L}}^{2}. \end{aligned}$$

We can take $k_{0}$ large enough such that $2^{-k_{0}/2}\|f\|_{\Lambda_{d(1/p-1)}^{L}}^{2}$ is small. This proves that $\|Q_{t}^{L} f\|_{T_{2}^{p,\infty}}<\infty$ and $\eta_{1}(f)=\eta_{2}(f)=\eta_{3}(f)=0$ follows from (23). Therefore $Q_{t}^{L} f\in T_{2,0}^{p,\infty}$.

Now we give the proof of (22). Set $B_{k}=B(x_{0}, 2^{k}r)$ and

$$f= \bigl(f-f(B_{1}) \bigr)\chi_{B_{1}}+ \bigl(f-f(B_{1}) \bigr)\chi _{(B_{1})^{c}}+f(B_{1})=\widetilde{f}_{1} + \widetilde{f}_{2} + f(B_{1}). $$

By Lemma 6, we have

24 $$\begin{aligned} \frac{1}{ \vert B \vert ^{2/p-1}} \int_{\widehat{B}} \bigl\vert Q^{L}_{t} \widetilde{f}_{1}(x) \bigr\vert ^{2} \frac{dx \,dt}{t} \leq& \frac{1}{ \vert B \vert ^{2/p-1}} \int_{B} \bigl\vert s_{L} \widetilde{f}_{1}(x) \bigr\vert ^{2} \,dx \\ =& \frac{1}{4 \vert B \vert ^{2/p-1}} \int_{B_{1}} \bigl\vert f(x)- f(B_{1}) \bigr\vert ^{2} \,dx \leq C \beta_{1}(f,B). \end{aligned}$$

By

$$\bigl\vert f(B_{k+1})- f(B_{1}) \bigr\vert \leq C \sum _{i=2}^{k+1} \vert B_{i} \vert ^{1/p-1} \frac{1}{ \vert B_{i} \vert ^{1/p-1/2}} \biggl( \int_{B_{i}} \bigl\vert f(x)-f(B_{i}) \bigr\vert ^{2}\,dx \biggr)^{1/2} $$

and Lemma 5(a), for $x \in B(x_{0},r)$,

$$\begin{aligned} \bigl\vert Q^{L}_{t} \widetilde{f}_{2}(x) \bigr\vert \leq& C \int_{\mathbb{R}^{d}} \frac{t}{ \vert x_{0}-y \vert ^{(d+1)}} \bigl\vert \widetilde {f}_{2}(y) \bigr\vert \,dy \\ \leq& C \int_{(B_{1})^{c}} \frac{t}{ \vert x_{0}-y \vert ^{(d+1)}} \bigl\vert f(y)-f(B_{1}) \bigr\vert \,dy \\ \leq& C \sum^{\infty}_{k=1} \frac{t}{(2^{k} r)^{d+1}} \biggl( \int_{B_{k+1} \setminus B_{k}} \bigl\vert f(y) - f(B_{k+1}) \bigr\vert \,dy \\ &{} + \bigl(2^{k} r\bigr)^{d} \bigl\vert f(B_{k+1})- f(B_{1}) \bigr\vert \biggr) \\ \leq& C \frac{t}{r^{1-d(1/p-1)}} \sum^{\infty}_{k=1} 2^{k(d(1/p-1)-1)}\biggl(\frac{1}{ \vert B_{k+1} \vert ^{1/p-1/2}}\biggl( \int _{B_{k+1}} \bigl\vert f-f(B_{k+1}) \bigr\vert ^{2} \,dy\biggr)^{1/2} \\ &{} +\sum_{i=2}^{k+1}\frac{1}{ \vert B_{i} \vert ^{1/p-1/2}} \biggl( \int _{B_{i}} \bigl\vert f-f(B_{i}) \bigr\vert ^{2} \,dy\biggr)^{1/2}\biggr) \\ \leq& C\frac{t}{r^{1-d(1/p-1)}}\sum^{\infty}_{k=1} 2^{k(d(1/p-1)-1)}(1+k)\beta_{k}(f,B)^{1/2}. \end{aligned}$$

Therefore

25 $$ \frac{1}{|B|^{2/p-1}} \int_{\widehat{B}} \bigl\vert Q^{L}_{t} \widetilde{f}_{2}(x) \bigr\vert ^{2} \frac{dx \,dt}{t} \leq C \sum^{\infty}_{k=1} 2^{k(d(1/p-1)-1)} \beta_{k}(f,B). $$

It remains to estimate the constant term. Assume first that $r< \rho(x_{0})$. Taking $k_{0}$ such that $2^{k_{0}}r< \rho(x_{0}) \leq 2^{k_{0}+1}r$, we have

$$\begin{aligned} \bigl\vert f(B_{1}) \bigr\vert \leq& \bigl\vert f(B_{k_{0}+1})- f(B_{1}) \bigr\vert + \bigl\vert f(B_{k_{0}+1}) \bigr\vert \\ \leq&C\sum_{i=2}^{k_{0}+1} \vert B_{i} \vert ^{1/p-1}\frac {1}{ \vert B_{i} \vert ^{1/p-1/2}} \biggl( \int_{B_{i}} \bigl\vert f-f(B_{i}) \bigr\vert ^{2} \,dy \biggr)^{1/2} \\ &{} + \vert B_{k_{0}+1} \vert ^{1/p-1}\frac{1}{ \vert B_{k_{0}+1} \vert ^{1/p-1/2}} \biggl( \int _{B_{k_{0}+1}} \vert f \vert ^{2} \,dy \biggr)^{1/2} \\ \leq& C \vert B_{k_{0}+1} \vert ^{1/p-1}(k_{0}+1) \beta_{k_{0}}^{1/2}(f,B). \end{aligned}$$

Note that $\rho(x) \sim\rho(x_{0})>r$ for any $x \in B(x_{0},r)$, by Lemma 5(c), we get

26 $$\begin{aligned}& \frac{1}{|B|^{2/p-1}} \int_{\widehat{B}} \bigl\vert Q^{L}_{t} \bigl( f(B_{1}) \mathbf{1} \bigr) (x) \bigr\vert ^{2} \frac{dx \,dt}{t} \\& \quad = \frac{ | f(B_{1}) |^{2}}{|B|^{2/p-1}} \int_{\widehat{B}} \biggl\vert \int_{\mathbb{R}^{d}} Q^{L}_{t}(x,y) \,dy \biggr\vert ^{2} \frac{dx \,dt}{t} \\& \quad \leq \frac{C | f(B_{1}) |^{2}}{|B|^{2/p-1}} \int_{\widehat{B}} \biggl( \frac{t}{\rho(x_{0})} \biggr)^{2} \frac{dx \,dt}{t} \\& \quad \leq C \frac{|B_{k_{0}+1}|^{2/p-2}}{|B|^{2/p-2}} ( 1+k_{0})^{2} \biggl( \frac{r}{\rho(x_{0})} \biggr)^{2}\beta_{k_{0}}(f,B) \\& \quad \leq C 2^{-2k_{0}(1-d(1/p-1))}(1+k_{0})^{2} \beta_{k_{0}}(f,B) \\& \quad \leq C 2^{-k_{0}(1-d(1/p-1))}\beta_{k_{0}}(f,B). \end{aligned}$$

For $r \geq\rho(x_{0})$, we have

$$\bigl\vert f(B_{1}) \bigr\vert \leq C |B_{1}|^{1/p-1} \frac{1}{|B_{1}|^{1/p-1/2}} \biggl( \int_{B_{1}} \bigl\vert f-f(B_{1}) \bigr\vert ^{2} \,dy \biggr)^{1/2}. $$

Note that $\rho(x) \leq Cr$ for any $x \in B(x_{0},r)$, again by Lemma 5(c),

27 $$\begin{aligned}& \frac{1}{|B|^{2/p-1}} \int_{\widehat{B}} \bigl\vert Q^{L}_{t} \bigl( f(B_{1})\mathbf{1} \bigr) (x) \bigr\vert ^{2} \frac{dx \,dt}{t} \\& \quad \leq\frac{ |f(B_{1}) |^{2}}{|B|^{2/p-1}} \int^{\infty}_{0} \int_{B} \biggl\vert \int_{\mathbb{R}^{d}} Q^{L}_{t}(x,y) \,dy \biggr\vert ^{2} \frac{dx \,dt}{t} \\& \quad \leq\frac{C | f(B_{1}) |^{2}}{|B|^{2/p-1}} \biggl( \int_{B} \int^{\rho(x)}_{0} \biggl( \frac{t}{\rho(x)} \biggr)^{2} \frac{dt}{t} \,dx + \int_{B} \int^{\infty}_{\rho(x)} \biggl( \frac{t}{\rho(x)} \biggr)^{-2} \frac{dt}{t} \,dx \biggr) \\& \quad \leq C \frac{|B_{1}|^{2/p-1}}{|B|^{2/p-1}}\beta_{1}(f,B)\leq 2^{d(1/p-1)-1} \beta_{1}(f,B). \end{aligned}$$

Then (22) follows from (24)–(27).

For the reverse, by Theorem 1, we get $f\in \Lambda_{d(1/p-1)}^{L}$ from $Q_{t}^{L}f\in T_{2,0}^{p,\infty}$. For any ball $B=B(x_{0},r)$,

$$\begin{aligned} \biggl( \int_{B} \bigl\vert f(x)-f(B) \bigr\vert ^{2} \,dx\biggr)^{1/2} =&\sup_{\operatorname{supp} g\subset B, \|g\|_{L^{2}(B)}\leq 1} \biggl\vert \int_{B}\bigl(f(x)-f(B)\bigr)g(x)\,dx \biggr\vert \\ =&\sup_{\operatorname{supp} g\subset B, \|g\|_{L^{2}(B)}\leq 1} \biggl\vert \int_{B}f(x) \bigl(g(x)-g(B)\bigr)\,dx \biggr\vert . \end{aligned}$$

Let $G(x)= (g(x)-g(B) )\chi_{B}$. Then, by Lemma 7, we obtain

$$\begin{aligned} \biggl\vert \int_{\mathbb{R}^{d}}f(x)G(x)\,dx \biggr\vert =&4 \biggl\vert \int_{\mathbb{R}^{d+1}_{+}}Q_{t}^{L}f(x) \bigl(Q_{t}^{L}g(x)-Q_{t}^{L}g(B) (x) \bigr)\frac{dx \,dt}{t} \biggr\vert \\ \leq&C \int_{\widehat{B_{2}}} \bigl\vert Q_{t}^{L}f(x) \bigr\vert \bigl\vert Q_{t}^{L}G(x) \bigr\vert \frac{dx \,dt}{t} \\ &{}+\sum_{k=2}^{\infty} \int_{\widehat{B_{k+1}}\setminus \widehat{B_{k}}} \bigl\vert Q_{t}^{L}f(x) \bigr\vert \bigl\vert Q_{t}^{L}G(x) \bigr\vert \frac{dx \,dt}{t} \\ =&E_{1}+\sum_{k=2}^{\infty}E_{k}. \end{aligned}$$

By Hölder’s inequality and Lemma 6, we have

28 $$\begin{aligned} E_{1} \leq& \biggl( \int_{\widehat{B_{2}}} \bigl\vert Q_{t}^{L}f(x) \bigr\vert ^{2}\frac{dx \,dt}{t} \biggr)^{1/2} \biggl\Vert \biggl( \int_{0}^{\infty}\bigl\vert Q_{t}^{L} \bigl(g-g(B) \bigr) (x) \bigr\vert ^{2}\frac{dt}{t} \biggr)^{1/2} \biggr\Vert _{L^{2}(B_{2})} \\ \leq&C \biggl( \int_{\widehat{B_{2}}} \bigl\vert Q_{t}^{L}f(x) \bigr\vert ^{2}\frac{dx \,dt}{t} \biggr)^{1/2}. \end{aligned}$$

Now, we estimate $E_{k}$. By Hölder’s inequality again, we have that

$$E_{k}\leq F_{k}\cdot I_{k}, $$

where

$$F_{k}= \biggl( \int_{\widehat{B_{k+1}}\setminus\widehat{B_{k}}} \bigl\vert Q_{t}^{L}f(x) \bigr\vert ^{2}\frac{dx\,dt}{t} \biggr)^{1/2} $$

and

$$I_{k}= \biggl( \int_{\widehat{B_{k+1}}\setminus\widehat{B_{k}}} \bigl\vert Q_{t}^{L}g(x)-Q_{t}^{L}g(B) (x) \bigr\vert ^{2}\frac{dx\,dt}{t} \biggr)^{1/2}. $$

When $r<\rho(x_{0})$, then $\int_{B}g(x)-g(B)\,dx=0$. Therefore, by Lemma 5(b),

$$\begin{aligned} \bigl\vert Q_{t}^{L}g(x)-Q_{t}^{L}g(B) (x) \bigr\vert =& \biggl\vert \int_{B} \bigl(Q_{t}^{L}(x,y)-Q_{t}^{L}(x,x_{0}) \bigr) \bigl(g(y)-g(B)\bigr)\,dy \biggr\vert \\ \leq&C \int_{B}\frac{t}{(t+|x-y|)^{d+1}}\frac{|x_{0}-y|}{t} \bigl\vert g(y)-g(B) \bigr\vert \,dy \\ \leq&C \int_{B}\frac{t}{(2^{k}r)^{d+1}}\frac{r}{t} \bigl\vert g(y)-g(B) \bigr\vert \,dy \\ \leq&C\frac{t}{(2^{k}r)^{d+1}}\frac{r}{t}\|g\|_{L^{1}(B)}\leq C |B|^{1/2}\frac{t}{(2^{k}r)^{d+1}}\frac{r}{t}. \end{aligned}$$

Therefore

$$ I_{k}^{2}\leq C|B| \int_{0}^{2^{k+1}r} \int_{B_{k+1}}\frac {t^{2}}{(2^{k}r)^{2d+2}} \biggl(\frac{r}{t} \biggr)^{2} \frac{dx \,dt}{t} \leq C|B|\frac{1}{(2^{k}r)^{d}}2^{-2k}. $$

It follows that

$$E_{k}\leq C|B|^{1/2}|B_{k}|^{-1/2}2^{-k} \biggl( \int_{\widehat{B_{k+1}}} \bigl\vert Q_{t}^{L}f(x) \bigr\vert ^{2}\frac{dx \,dt}{t} \biggr)^{1/2}. $$

When $r\geq\rho(x_{0})$, we have $\rho(y)\leq C r$ for $y\in B(x_{0}, r)$. Then, by Lemma 5(a),

$$\begin{aligned} \bigl\vert Q_{t}^{L}g(x)-Q_{t}^{L}g(B) (x) \bigr\vert =& \biggl\vert \int_{B}Q_{t}^{L}(x,y)g(y)\, dy \biggr\vert \\ \leq&C \int_{B}\frac{t}{(2^{k}r)^{d+1}}\frac{\rho(y)}{t} \bigl\vert g(y) \bigr\vert \,dy \\ \leq&C\frac{t}{(2^{k}r)^{d+1}}\frac{r}{t}\|g\|_{L^{1}(B)}\leq C |B|^{1/2}\frac{t}{(2^{k}r)^{d+1}}\frac{r}{t}. \end{aligned}$$

Then we can get

29 $$ E_{k}\leq C|B|^{1/2}|B_{k}|^{-1/2}2^{-k} \biggl( \int_{\widehat{B_{k+1}}} \bigl\vert Q_{t}^{L}f(x) \bigr\vert ^{2}\frac{dx\,dt}{t} \biggr)^{1/2}. $$

By (28) and (29), we know

$$\begin{aligned}& \frac{1}{ \vert B \vert ^{1/p-1/2}} \biggl( \int_{B} \bigl\vert f(x)-f(B) \bigr\vert ^{2}\, dx \biggr)^{1/2} \\& \quad \leq \frac{C}{ \vert B \vert ^{1/p-1}}\sum_{k=1}^{\infty}2^{-k} \vert B_{k} \vert ^{-1/2} \biggl( \int_{\widehat{B_{k}}} \bigl\vert Q_{t}^{L}f(x) \bigr\vert ^{2}\frac{dx \,dt}{t} \biggr)^{1/2} \\& \quad = C\sum_{k=1}^{\infty}2^{-k} \frac{ \vert B_{k} \vert ^{1/p-1}}{ \vert B \vert ^{1/p-1}}\frac {1}{ \vert B_{k} \vert ^{1/p-1/2}} \biggl( \int_{\widehat{B_{k}}} \bigl\vert Q_{t}^{L}f(x) \bigr\vert ^{2}\frac{dx \,dt}{t} \biggr)^{1/2} \\& \quad \leq C\sum_{k=1}^{\infty}2^{-k(1-\alpha)} \sigma_{k}(f,B), \end{aligned}$$

where

$$\sigma_{k}(f,B)=\frac{1}{|B_{k}|^{1/p-1/2}} \biggl( \int_{\widehat {B_{k}}} \bigl\vert Q_{t}^{L}f(x) \bigr\vert ^{2}\frac{dx \,dt}{t} \biggr)^{1/2}. $$

Then we can get $\gamma_{1}(f)=\gamma_{2}(f)=\gamma_{3}(f)=0$ as the proof of the first part of this theorem. Therefore $f\in\lambda_{\alpha}^{L}$ and the proof of Theorem 2 is completed. □

## Conclusions

This paper defines a new version of Carleson measure associated with Hermite operator, which is adapted to the operator *L*. Then, we characterize the dual spaces and predual spaces of the Hardy spaces $H_{L}^{p}(\mathbb {R}^{d})$ associated with *L*. The main results of this paper are the central problems in harmonic analysis, which can be used in PED or geometry widely.
